# The Growth of SGC-7901 Tumor Xenografts Was Suppressed by Chinese Bayberry Anthocyanin Extract through Upregulating *KLF6* Gene Expression

**DOI:** 10.3390/nu8100599

**Published:** 2016-09-27

**Authors:** Yue Wang, Xia-nan Zhang, Wen-hua Xie, Yi-xiong Zheng, Jin-ping Cao, Pei-rang Cao, Qing-jun Chen, Xian Li, Chong-de Sun

**Affiliations:** 1 Laboratory of Fruit Quality Biology/The State Agriculture Ministry Laboratory of Horticultural Plant Growth, Development and Quality Improvement, Zhejiang University, Zijingang Campus, Hangzhou 310058, China; fruit@zju.edu.cn (Y.W.); xiananzhang@zju.edu.cn (X.-n.Z.); 21516063@zju.edu.cn (W.-h.X.); xianli@zju.edu.cn (X.L.); 2 Department of Surgery, Second Affiliated Hospital, School of Medicine, Zhejiang University, Hangzhou 310009, China; zyx_xxn@126.com; 3 Taizhou Academy of Agricultural Sciences, Linhai 317000, China; caojinpingabc@126.com; 4 State Key Laboratory of Food Science and Technology, School of Food Science and Technology, Jiangnan University, Wuxi 214122, China; prcao@jiangnan.edu.cn; 5 National Light Industry Food Quality Inspection Hangzhou Station, Hangzhou 310009, China; chenqj@zzytech.com

**Keywords:** Chinese bayberry, anthocyanin, SGC-7901 cell, tumor xenograft, *KLF6* gene

## Abstract

To investigate the antitumor effect of anthocyanins extracted from Chinese bayberry fruit (*Myrica rubra* Sieb. et Zucc.), a nude mouse tumor xenograft model was established. Treatments with C3G (cyanidin-3-glucoside, an anthocyanin) significantly suppressed the growth of SGC-7901 tumor xenografts in a dose-dependent manner. Immunohistochemical staining showed a significant increase in p21 expression, indicating that the cell cycle of tumor xenografts was inhibited. qPCR screening showed that C3G treatment up-regulated the expression of the *KLF6* gene, which is an important tumor suppressor gene inactivated in many human cancers. Western blot showed that C3G treatments markedly increased KLF6 and p21 protein levels, inhibited CDK4 and Cyclin D1 expression, but did not notably change the expression of p53. These results indicated that KLF6 up-regulates p21 in a p53-independent manner and significantly reduces tumor proliferation. This study provides important information for the possible mechanism of C3G-induced antitumor activity against gastric adenocarcinoma in vivo.

## 1. Introduction

Anthocyanins are the most abundant water-soluble pigment found in fruit, vegetables and beans. It has been well established that anthocyanins from different sources exhibit multiple functional properties including antioxidant [1], anticancer [2], anti-obesity [3] and anti-diabetic effects [4]. It has been demonstrated in our laboratory that the proliferation of human SGC-7901, BGC-823 and AGS gastric cancer cells in vitro was inhibited by anthocyanins from Chinese bayberry fruit [5].

Gastric cancer, also called stomach cancer, is the fourth most frequently diagnosed cancer in humans and the third leading cause of cancer death worldwide [6]. It ranks second as the most frequently diagnosed cancer and is the leading cause of cancer death in China today, second only to lung cancer [7]. Further epidemiological and experimental studies showed that diet pattern variations play an important role in the etiology of gastric cancer [8,9], and a reverse association between fruit intake and gastric cancer risk has been widely reported [10,11,12,13].

Chinese bayberry (*Myrica rubra* Sieb. et Zucc.) is a subtropical native Chinese fruit with high nutrient and health values. The red-colored Chinese bayberry pulp is a rich source of anthocyanins, especially cyanidin-3-glucoside (C3G) [14], which has been well characterized as having anticancer activity in vitro [15] and in vivo [16]. Previous studies have demonstrated that the in vitro anticancer activities of anthocyanins are exerted through mechanisms of promotion of apoptosis [17], inhibition of cell cycle [18] and cell invasion [5,19]. However, the antitumor effects of C3G in vivo have been less clearly demonstrated.

Krüppel-like transcription factor 6 (*KLF6*) is a novel tumor suppressor gene and is involved in the pathogenesis of many cancers [20]. In addition to gastric cancer [21], functional inactivation of *KLF6* was observed in a number of other human cancers including prostate [20], colorectal [22,23], ovarian [24], liver [25,26], and breast cancer [27]. With different cell types and contexts, *KLF6* exhibits growth inhibition activity through several major cancer pathways such as p53-independent up-regulation of p21 [20], disruption of Cyclin D1 and CDK4 interaction [28] and induction of apoptosis [23]. Although the tumor-suppressing activity of the *KLF6* gene is well known, it has not been reported whether natural nutrients such as anthocyanins could affect *KLF6* expression.

In this study, it was first discovered that C3G, a major component of anthocyanins from Chinese bayberry, could suppress the growth of SGC-7901 tumor xenografts through up-regulating *KLF6* gene expression.

## 2. Materials and Methods

### 2.1. Materials and Chemicals

Chinese bayberry (*Myrica rubra* Sieb. et Zucc c.v. Dongkui) fruits were harvested at commercial maturity from Xianju County, Zhejiang Province, China. The SGC-7901 gastric cancer cell line was obtained from the Department of Surgery, Second Affiliated Hospital, School of Medicine, Zhejiang University.

C3G standards were purchased from Sigma-Aldrich Co. LLC (Shanghai, China). RIPA (Radio Immunoprecipitation Assay) lysis buffer was purchased from the Beyotime Institute of Biotechnology (Hangzhou, China). Anti-KLF6, anti-caspase3, anti-p21, anti-p53, anti-Cyclin D1, anti-CDK4 antibodies were obtained from Proteintech (Chicago, IL, USA). All the other reagents were of analytical grade and purchased from Sinopharm Chemical Reagent Co., Ltd. (Shanghai, China). Double-distilled water (ddH_2_O) was used in all experiments. All samples for HPLC (High Performance Liquid Chromatography) analyses were filtered through a 0.22 μm membrane before injection.

### 2.2. Purification and Identification of C3G from Chinese Bayberry Fruit

Fifty grams of fresh Chinese bayberry fruit pulp was extracted ultrasonically with 80% aqueous methanol (1% formic acid) in a material-to-solvent ratio of 1:10 (*w*/*v*) three times. The supernatants from three extractions were combined and evaporated under reduced pressure at 36 °C to remove the methanol. A phenolic-rich extract (PRE) was obtained by solid-phase extraction (SPE) using a Sep-pak C_18_ cartridge (12 cc, 2 g sorbent; Waters Corp., Milford, MA, USA). Sugars and organic acids were removed by eluting with ddH_2_O. Phenolics were eluted with 10% aqueous methanol after eluting with 5% aqueous methanol.

The purity of C3G was determined by HPLC (2695 pump, 2996 diode array detector; Waters Corp., Milford, MA, USA) coupled with a Waters SunFire C_18_ analytical column (4.6 × 250 mm) at a column temperature of 25 °C. The HPLC analyses were performed as per previously published procedures [29] with some modifications. The mobile phase consisted of 0.1% (*v*/*v*) formic acid in water (eluent A) and of acetonitrile: 0.1% formic acid (1:1, *v*/*v*) (eluent B). The gradient program was as follows: 0 to 40 min, 10% to 38% of B; 40 to 60 min, 38% to 48% of B; 60 to 70 min, 48% to 100% of B; 70 to 75 min, 100% to 10% of B; 75 to 80 min, 10% of B. Then 10 μL samples were injected and were detected from 200 to 600 nm. Anthocyanins were calculated as C3G equivalent at 515 nm.

LC-MS (Liquid Chromatograph-Mass Spectrometer) experiments were performed according to our previous publication [5]. Briefly, an Agilent 6430 Triple Quadrupole LC/MS system (Agilent Technologies Inc., Santa Clara, CA, USA) was used for LC-MS. Multiple reaction monitoring (MRM) was used for analytical identification and electrospray ionization (ESI) was in positive mode. The operation conditions were as follows: capillary 4000 V, nebulizer 35 psi, dry gas flow rate 9 L/min at 350 °C. An Agilent MassHunter Workstation was used to carry out data acquisition and processing.

The ^1^H-NMR (Hydrogen-Nuclear Magnetic Resonance) (500.18 MHz) spectra were obtained on a Bruker Avance 500 instrument (Bruker Biospin, Fallanden, Switzerland). Extracts (15 mg) were dissolved in 0.5 mL deuterated methanol (CD_3_OD) in a 5 mm ф tube at variable temperatures; *δ* (parts per million) and the coupling constants (*J*) in Hertz were presented as chemical shifts.

### 2.3. Animal and Tumor Xenograft Studies

Balb/c nude mice (weighing 19–21 g) were used for building a model and were maintained at 23–25 °C and 50%–60% humidity in the Laboratory Animal Center of Zhejiang University (Hangzhou, China). Balb/c-nu mice were randomly allocated to four groups: a model group (drinking water), low-dose group (C3G, 25 mg·kg^−1^·bw^−1^·day^−1^), high-dose group (C3G, 125 mg·kg^−1^·bw^−1^·day^−1^) and positive control group (tegafur, 10 mg·kg^−1^·bw^−1^·day^−1^) (three mice in each group). Mice were s.c. injected with 2 × 10^6^ SGC-7901 cells on the right groin and were housed under a regular 12 h light/12 h dark cycle. After 18 days, the mice were sacrificed for the assay of tumorigenicity (e.g., body weight and tumor volume). All experiments were carried out in accordance with the ethical guidelines of the Animal Experimentation Committee in the College of Medicine, Zhejiang University. Our experiment ethic approval code is ZJU20160443.

### 2.4. Immunohistochemical Staining

The tumor tissues were removed from each mouse and the samples were subsequently fixed in 4% (*v*/*v*) paraformaldehyde/PBS (Phosphate Buffer Saline) and embedded in paraffin for staining. The paraffin sections were deparaffinized in xylene, rehydrated in a 10 mM citrate buffer (pH 6.0), and heated in a microwave oven for 15 min to restore the antigens. To suppress endogenous peroxidase within the tissues, the samples were treated with 3% peroxide for 5 min and then with a blocking solution for 30 min. Slides were incubated with the primary antibody and secondary antibody in a humid chamber for 60 min. Tissue staining was visualized with a 3,3′-diaminobenzidine substrate chromogen solution and the images were taken by using a microscope set (Zeiss, Germeny) at a 200× magnification.

### 2.5. Quantitative Real-Time PCR and Western Blot Assay

Total RNA was isolated from tumor tissues using Trizol reagent (Invitrogen, Waltham, MA, USA) according to the manufacturer’s protocol. Equal amounts of total RNA (1.0 μg) were used to synthesize cDNA with the iScriptTM cDNA Synthesis kit (Bio-Rad, Hercules, CA, USA). Quantitative real-time PCR was performed in triplicate using a SYBR Green Master I kit (Roche, Basel, Switzerland) and the LightCycler480 real-time PCR System (Roche, Basel, Switzerland). Gene-specific primers were used as mentioned in Table 1. The RNA quality was detected by OD_260_/OD_280_ and gel electrophoresis. The fold change of the treatment group versus control group for each target gene was calculated using the 2^−ΔΔCt^ method and was evaluated as the effect of treatment on relative gene expression. Expression was normalized against expression of the housekeeping gene β-*actin*.

Tumor tissues were lysed using RIPA buffer containing 1% PMSF (Phenylmethanesulfonyl Fluoride), and protein inhibitor (cOmplete mini, Roche, Los Angeles, CA, USA). The protein concentration was determined using the Pierce BCA Protein Assay Kit (Thermo Scientific, Waltham, MA, USA). The concentration was adjusted to 1 μg/μL using PBS and 5× loading buffer. Equal amounts (40 μg) of protein were separated on a 10% SDS (Sodium Dodecyl Sulfate)-polyacrylamide gel and transferred onto PVDF (Polyvinylidene Fluoride) membranes. After blocking the membrane with 5% nonfat dried milk for 1.5 h, the protein abundance was detected with antibodies against KLF6 (1:1000 dilutions), p21 (1:1000 dilutions), p53 (1:1000 dilutions), Cyclin D1 (1:1000 dilutions), CDK4.

(1:1000 dilutions), followed by incubation with peroxide-conjugated anti-rabbit immunoglobulin. β-*actin* was used as a loading control.

### 2.6. Statistics

Statistical analyses were carried out using SPSS version 19.0 (IBM, Armonk, NY, USA). Data were analyzed by one-way ANOVA. Multiple comparison between the groups was performed using the LSD (Least Significant Difference) method. OriginPro 8.0 software packages (OriginLab Corporation, Northampton, MA, USA) was used for plotting the experimental data. Values were expressed as the mean ± standard deviation.

## 3. Results

### 3.1. Purification of C3G Extracted from Chinese Bayberry

**Figure 1:**
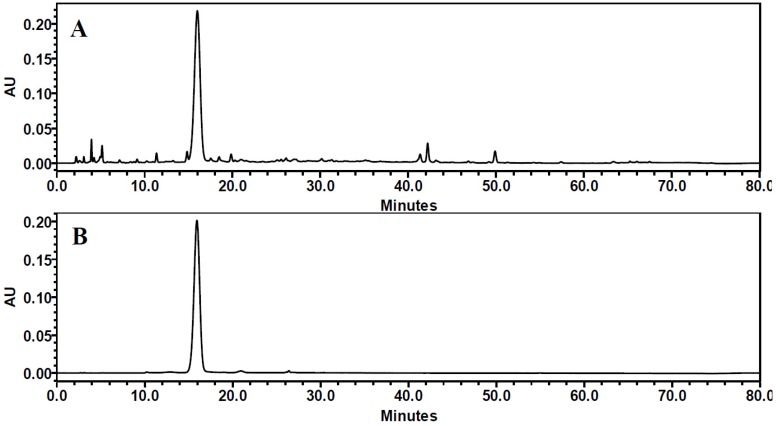

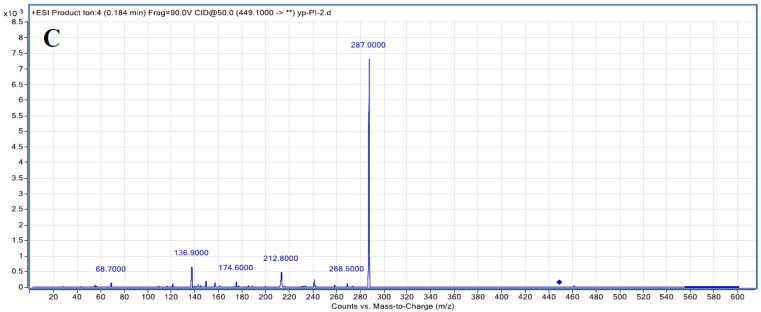
HPLC chromatograms of Chinese bayberry pulp (**A**) and purified C3G from Chinese bayberry (**B**) (λ = 280 nm) and LC-MS^2^ spectrum of purified C3G (**C**).

The identification and quantification of C3G extracted and purified from Chinese bayberry was accomplished by HPLC, LC-MS and ^1^H-NMR (Figure 1). HPLC identification was carried out according to the retention time and peak area compared with those of standard C3G. HPLC analysis of the identified anthocyanin compounds showed that the retention time of cyanidins-3-glucoside was 15.6 to 17.9 min. The HPLC chromatograms of Chinese bayberry pulp showed impurity peaks (Figure 1A), while purified C3G extracts did not have any other significant peaks (Figure 1B). The purity was determined with a standard curve chart and C3G with a purity of 88.81% was achieved.

Further identification of C3G was confirmed by LC-MS (Figure 1C) and ^1^H-NMR (Table 2). Among all the LC-MS^2^ products, ions at *m*/*z* 287.0 were present in great abundance which was related to the loss of one hexose ([M − 162]^+^) molecule, furnishing the cyanidin aglycone, the main body structure of C3G. The ions at *m*/*z* 136.9 and 240.6 resulted from the ring-cross cleavage of cyanidin. The ^1^H-NMR spectrum showed the presence of one cyaniding nucleus and one hexose (Table 2).

Therefore, all HPLC, LC-MS and ^1^H-NMR data confirmed the purified extract was C3G.

### 3.2. C3G Suppressed the Growth of SGC-7901 Tumor Xenografts

**Figure 2:**
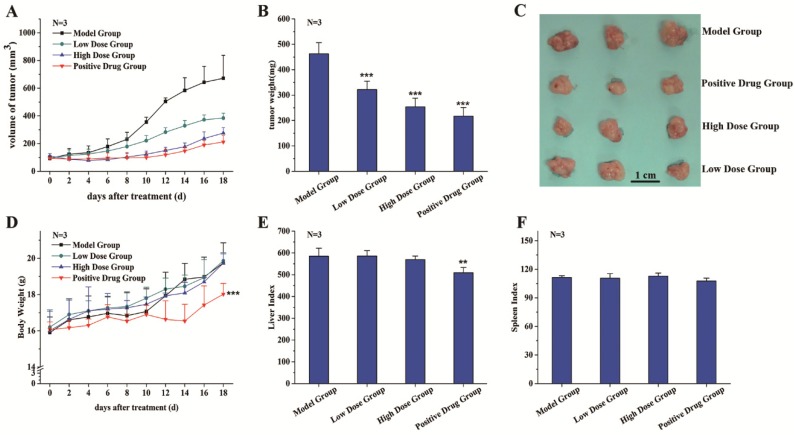
Effects of purified C3G on tumorigenesis in vivo. (**A**) The volumes of tumors were monitored at the indicated times; (**B**) tumor weights were measured after rats were sacrificed; (**C**) photographs of individual tumor xenografts removed from mice; (**D**) the body weights were monitored every alternate day after treatments; (**E**) the liver index and (**F**) the spleen index. The index was calculated as liver weight (mg)/body weight (g); and spleen weight (mg)/body weight (g), respectively. Values are mean ± SEM of three mice. ** *p* < 0.01, *** *p* < 0.001.

A tumor xenograft model was established to study the inhibition effects of C3G on tumor growth in vivo. Through preliminary tests (data not shown), we chose 25 mg·kg^−1^·bw^−1^·day^−1^ and 125 mg·kg^−1^·bw^−1^·day^−1^ as our C3G treatment doses and selected 18 days as the duration of this experiment. The effects of C3G on tumor size were monitored every alternate day. After eight days of cancer cell injection treatment, the average tumor volume in the control group began to appear significantly different from that of other treatment groups, reaching up to 230.8 mm^3^ (Figure 2A). At 18 days before the sacrifice, the average tumor volume in the control group had increased to 771.5 mm^3^, while the C3G treatment groups with low dose and high dose grew to 384.1 mm^3^ and 276.0 mm^3^, respectively, compared to the tegafur-treated group (positive control), which was 211.7 mm^3^ (Figure 2A).

The inhibitory effect of C3G on tumor growth was evaluated based on tumor xenograft size when tumor tissues were removed and weighed after sacrifice. The tumor weight and size of treatment groups were significantly lower than those of the control group (Figure 2B,C). Inhibition rates of the low C3G dose group, the high dose group and the drug group were 30.4%, 45.1% and 53.0%, respectively.

Experimental results showed no significant changes in body weight, liver index and spleen index between the control group and C3G treatment groups (Figure 2D–F), indicating that C3G extracted from Chinese bayberry has little toxicity to mice. However, compared with the control group and C3G treatment groups, the body weight and liver index of the tegafur group was significant decreased (Figure 2D,E), showing the positive drug had some toxic effects or side effects on the liver and whole body of mice.

### 3.3. C3G Inhibited the Cell Cycle of SGC-7901 Tumor Xenografts

**Figure 3:**
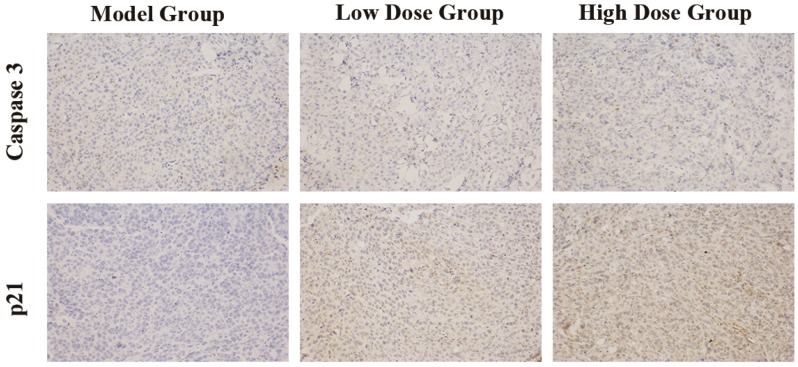
Caspase 3 and p21 immunochemical staining of tumor xenografts sections (200× magnification) (*n* = 3).

Expressions of caspase 3 and p21 in tumor tissues were determined by immunohistochemistry staining. The negative cellular nuclei were stained blue, while the positive nuclei were stained dark brown. Immunohistochemical studies showed clear positive p21 staining in cell nuclei in C3G treatment groups with a dose-dependent manner. In contrast, the positive caspase 3 staining, which appeared in the cytoplasm, showed no marked change among different treatments (Figure 3). These results indicated that anthocyanin C3G from Chinese bayberry could suppress the growth of tumor xenografts by inhibiting the cell cycle but with less induction of apoptosis.

### 3.4. Gene Expression Analysis

**Figure 4:**
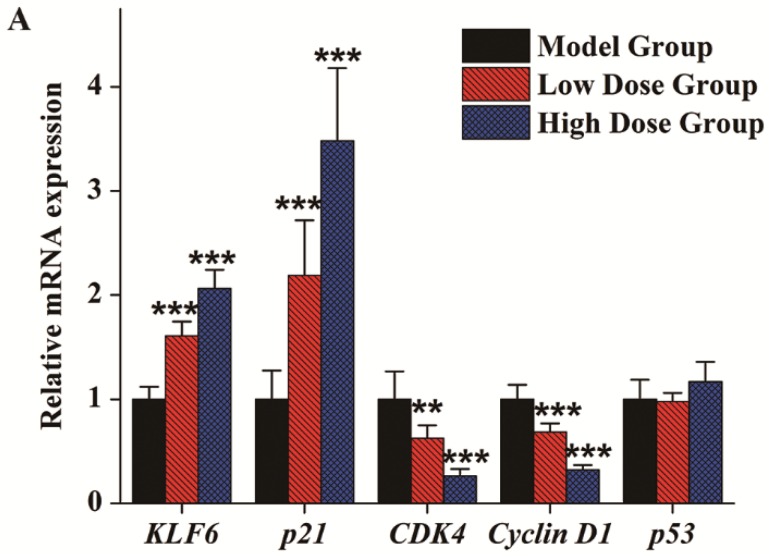

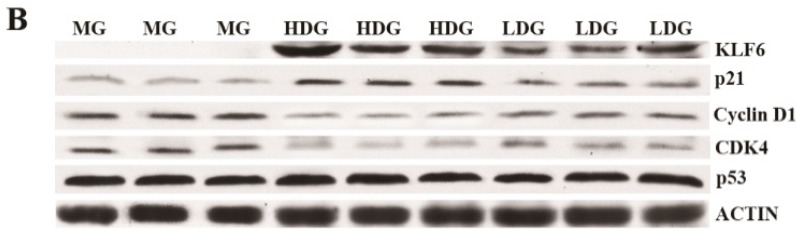
Effects of purified C3G on gene expression and protein expression in tumor xenografts. (**A**) Relative mRNA expression of *KLF6*, *p21*, *CDK4*, *Cyclin D1*, *p53* after C3G treatments; (**B**) protein expression of KLF6, p21, CDK4, Cyclin D1, p53 after C3G treatments. Values are mean ± SEM of measurements from three mice. ** *p* < 0.01, *** *p* < 0.001.

To elucidate the in vivo mechanism of C3G inhibition of the cell cycle in SGC-7901 cell tumor xenografts, the effects of C3G on expression of cancer-related genes, including *KLF6*, *p21*, *Cyclin D1*, *CDK4* and *p53*, were further evaluated. Both mRNA levels, measured by quantitative real-time PCR, and protein levels, measured by Western blot, were analyzed to determine these effects. As indicated in Figure 4A, results showed that the *KLF6* and *p21* genes were up-regulated in a dose-dependent manner in C3G treatment groups. The relative mRNA expression of *KLF6* in the low dose and high dose groups was about 1.61- and 2.06-fold higher than in the control group. Similarly, *p21* was up-regulated 2.19- and 3.48-fold in the low dose group and high dose group, respectively. Both *Cyclin D1* and *CDK4* gene expressions in C3G treatment groups were inhibited significantly. In comparison with the control group (Figure 4A), *Cyclin D1* gene expression was decreased 0.62- and 0.26-fold and the *CDK4* gene was down-regulated 0.69- and 0.32-fold, respectively. However, there were no obvious changes in the expression of *p53*, the master tumor suppressor gene, between the treatment groups and the control (Figure 4A). The protein expressions of various genes as measured by Western blot analysis were in accordance with their qPCR analysis (Figure 4B). KLF6 and p21 protein levels were significantly increased, indicating the critical role of KLF6 in the tumor xenograft suppression in vivo. However, the p53 protein did not change significantly, whereas CDK4 and Cyclin D1 proteins were decreased with C3G treatments.

## 4. Discussion

A nude mouse tumor xenograft model was established to study the in vivo anticancer effect of high-purity anthocyanin C3G from Chinese bayberry. Results showed that C3G inhibited the growth of SGC-7901 cell tumor xenografts in a dose-dependent manner. The inhibition rates of low dose and high dose groups were 30.4% and 45.1%, respectively. Compared with the positive drug tegafur treatment, the C3G treatment did not show any toxic effects or other side effects. Immunohistochemical staining indicated that the tumor-suppressing effect was due to cell cycle inhibition but lower apoptosis induction. Through qPCR and Western blot, it was established that C3G treatment could up-regulate *KLF6* gene expression and the downstream effector *p21* in a *p53*-independent manner. As a consequence, it is concluded that C3G activity on tumor growth inhibition was probably through KLF6-mediated p21 induction, by disruption of the Cyclin D1 and CDK4 interaction, thus blocking the cell cycle.

The Krüppel-like family (KLF) of transcription factors is characterized by three contiguous C2H2-type zinc finger motifs at the carboxy terminus which comprise the DNA-binding domain [30,31]. *KLF6* is a member of the KLF family that consists of four exons and encodes a nuclear core promoter element-binding protein [32]. *KLF6* is a tumor suppressor based on its inactivation and somatic mutations in a variety of cancers such as prostate [20], gastric [21], ovarian [24], breast [27], liver [25,26], and colorectal cancer [22,23]. *KLF6*’s growth-suppressive activity is linked to p53-independent transactivation of p21 [20] and inhibition of the Cyclin D1/CDK4 complex [28]. This study could be the first report that *KLF6* can be up-regulated by anthocyanin C3G extracted from Chinese bayberry, thus reducing the mouse tumor growth burden.

In vitro effects of C3G are well studied and it is known that C3G can suppress tumor cell proliferation by various mechanisms such as apoptosis induction [17], cell cycle inhibition [18], peroxidation inhibition [33] and migration inhibition [5,19], etc. Several signaling pathways have been implicated, such as inhibiting Ras signaling [16], down-regulating the expressions of CDKs [34], abolishing ethanol-mediated p130^Cas^/JNK interaction [19], elevating the Bax/Bcl-2 ratio [17], and inducing signaling by p38/p53 and c-jun [35]. Mulberry anthocyanins containing 46.13% of cyanidine-3-gluoside induce apoptosis in gastric cancer cell AGS by up-regulating *p53* and other apoptosis-mediated gene expression [35]. However, the in vivo experiment from this study did not show any significant change of *p53* expression in tumor tissues, either at the mRNA or protein level, illustrating that the mechanism of the antitumor effect of C3G in vitro and in vivo might be different. There have been multiple reports about antitumor effects of anthocyanins extracted from natural products. Baked purple-fleshed potato reduced the colon CSCs number via induction of apoptosis [36]. Anthocyanins extracted from Korean wild berry, Meoru, could inhibit hepato-carcinoma cell metastasis via the AMPK pathway [37]. Delphinidin-3-glucoside suppressed breast carcinogenesis in vivo through decreasing HOX transcript antisense RNA (HOTAIR) transcript [38]. Pomegranate polyphenolics showed cytotoxicity in vitro and in vivo through the PI3K/AKT pathway [39]. A diet containing anthocyanins from black raspberries inhibited NMBA-induced rat esophagus tumor development by reducing NF-κB and COX-2 expression [40]. Anthocyanin-rich extract from roselle could inhibit *N*-Nitrosomethylurea–induced leukemia in vivo [41]. A diet with anthocyanin-enriched potato P40 prevented rats from colorectal cancer [42]. Furthermore, although its tumor inhibition capacities are clearly indicated [16,35,43,44], the reported in vivo C3G antitumor mechanisms are nuanced in different animal models and different purities of C3G. However, reports expatiating the mechanism of C3G suppressing tumor growth in vivo are limited [16,35,43,44], which is partly due to the difficulty of separating and purifying high-purity C3G.

In this study, by using high-purity C3G and a tumor xenograft model, it was demonstrated that C3G treatment could up-regulate *KLF6* gene expression and *KLF6* exhibited tumor growth–suppressing activity by cell cycle inhibition with a p53-independent up-regulation of p21 and disrupted the Cyclin D1 and CDK4 interaction. However, further progress could be made by studying the oral administration of C3G, its metabolism and digestion in mice to investigate the target points of C3G. Since the *KLF6* gene belongs to the transcription factor Krüppel-like family, the antitumor function of other KLF family members could also be studied.

## 5. Conclusions

In conclusion, C3G treatment showed in vivo antitumor effects in dose-dependent manner, where significantly tumor growth inhibition was observed in nude mouse xenograft model. C3G treatments also showed no toxic effects or other side effects compared with the positive drug tegafur treatment. The tumor-suppressing effect was due to cell cycle inhibition but lower apoptosis induction through Immunohistochemical staining test. Further q-PCR and Western blot results showed that C3G treatment could up-regulate the *KLF6* gene expression and the downstream effector *p21* in a *p53*-independent manner. These results might provide important information concerning the possible mechanism of C3G-induced antitumor activity against gastric adenocarcinoma in vivo and shed light on the potential application of food anthocyanins in cancer prevention.

## Figures and Tables

**Table 1:** Primer sequences used in quantitative real-time PCR.

Gene	Forward Primer (5′ to 3′)	Reverse Primer (3′ to 5′)
*p53*	AGGCCTTGGAACTCAAGGAT	CCCTTTTTGGACTTCAGGTG
*KLF6*	GACAGCTCCGAGGAACTTTCT	CACGCAACCCCACAGTTGA
*P21*	TGGAGACTCTCAGGGTCGAAAA	GGCGTTTGGAGTGGTAGAAATCT
*CDK4*	ACAGTTCGTGAGGTGGCTTTA	TCAGATCCTTGATCGTTTCG
*Cyclin D1*	GAACACGGCTCACGCTTACC	GCCCAGACCCTCAGACTTGC
β-*actin*	TGACGTGGACATCCGCAAAG	CTGGAAGGTGGACAGCGAGG

**Table 2:** ^1^H spectral data (ppm) for purified C3G.

Cyanidin-3-Glucoside	^1^H
***Aglycone***	
4	9.02 (s)
6	6.63 (d, *J* = 2.5 Hz)
8	7.06 (d, *J* = 2 Hz)
2′	8.08 (s)
5′	7.10 (s)
6′	8.26 (s)
***Glucose***	
1″	5.30 (d, *J* = 16 Hz)
2″	3.70 (m)
3″	3.66 (br, s)
4″	3.54 (m)
5″	3.63 (s)
6″A	4.02 (s)
6″B	3.83 (s)

## Abbreviations

The following abbreviations are used in this manuscript:

C3G	Cyanidin-3-glucoside
CDK4	Cyclin-dependent kinase 4
RIPA	Radio Immunoprecipitation Assay
HPLC	High Performance Liquid Chromatography
LC-MS	Liquid Chromatograph-Mass Spectrometer
^1^H-NMR	Hydrogen-Nuclear Magnetic Resonance
PBS	Phosphate Buffer Saline
PMSF	Phenylmethanesulfonyl Fluoride
SDS	Sodium Dodecyl Sulfate
PVDF	Polyvinylidene Fluoride
KLF6	Krueppel-Like Factor 6
LSD	Least Significant Difference
CSCs	Cancer stem cells

## References

[1] Bräunlich M., Slimestad R., Wangensteen H., Brede C., Malterud K.E., Barsett H. (2013). Extracts, anthocyanins and procyanidins from aronia melanocarpa as radical scavengers and enzyme inhibitors. Nutrients.

[2] Chen P.N., Chu S.C., Chiou H.L., Kuo W.H., Chiang C.L., Hsieh Y.S. (2006). Mulberry anthocyanins, cyanidin 3-rutinoside and cyanidin 3-glucoside, exhibited an inhibitory effect on the migration and invasion of a human lung cancer cell line. Cancer Lett..

[3] Meydani M., Hasan S.T. (2010). Dietary polyphenols and obesity. Nutrients.

[4] Zhang X., Lv Q., Jia S., Chen Y., Sun C., Li X., Chen K. (2016). Effects of flavonoids-rich chinese bayberry (*Morella rubra* Sieb. et Zucc.) fruits extract on regulating glucose and lipids metabolism in diabetic KK-Ay mice. Food Funct..

[5] Sun C., Zheng Y., Chen Q., Tang X., Jiang M., Zhang J., Li X., Chen K. (2012). Purification and anti-tumour activity of cyanidin-3-*O*-glucoside from Chinese bayberry fruit. Food Chem..

[6] Torre L.A., Bray F., Siegel R.L., Ferlay J., Lortet-Tieulent J., Jemal A. (2015). Global cancer statistics, 2012. CA Cancer J. Clin..

[7] Chen W., Zheng R., Baade P.D., Zhang S., Zeng H., Bray F., Jemal A., Yu X.Q., He J. (2016). Cancer statistics in China, 2015. CA Cancer J. Clin..

[8] Liu C., Russell R.M. (2008). Nutrition and gastric cancer risk: An update. Nutr. Rev..

[9] Song P., Wu L., Guan W. (2015). Dietary nitrates, nitrites, and nitrosamines intake and the risk of gastric cancer: A meta-analysis. Nutrients.

[10] Lunet N., Lacerda-Vieira A., Barros H. (2005). Fruit and vegetables consumption and gastric cancer: A systematic review and meta-analysis of cohort studies. Nutr. Cancer.

[11] Nouraie M., Pietinen P., Kamangar F., Dawsey S.M., Abnet C.C., Albanes D., Virtamo J., Taylor P.R. (2005). Fruits, vegetables, and antioxidants and risk of gastric cancer among male smokers. Cancer Epidem. Biomark..

[12] González C.A., Pera G., Agudo A., Bueno-de-Mesquita H.B., Ceroti M., Boeing H., Schulz M., Del Giudice G., Plebani M., Carneiro F. (2006). Fruit and vegetable intake and the risk of stomach and oesophagus adenocarcinoma in the European prospective investigation into cancer and nutrition (EPIC–EURGAST). Int. J. Cancer.

[13] Vauzour D., Rodriguez-Mateos A., Corona G., Oruna-Concha M.J., Spencer J.P. (2010). Polyphenols and human health: Prevention of disease and mechanisms of action. Nutrients.

[14] Zhang W., Li X., Zheng J., Wang G., Sun C., Ferguson I.B., Chen K. (2008). Bioactive components and antioxidant capacity of Chinese bayberry (*Myrica rubra* Sieb. and Zucc.) fruit in relation to fruit maturity and postharvest storage. Eur. Food Res. Technol..

[15] Sorrenti V., Vanella L., Acquaviva R., Cardile V., Giofre S., Di Giacomo C. (2015). Cyanidin induces apoptosis and differentiation in prostate cancer cells. Int. J. Oncol..

[16] Fukamachi K., Imada T., Ohshima Y., Xu J., Tsuda H. (2008). Purple corn color suppresses ras protein level and inhibits 7,12-dimethylbenz[a]anthracene-induced mammary carcinogenesis in the rat. Cancer Sci..

[17] Shih P.H., Yeh C.T., Yen G.C. (2005). Effects of anthocyanidin on the inhibition of proliferation and induction of apoptosis in human gastric adenocarcinoma cells. Food Chem. Toxicol..

[18] Malik M., Zhao C.W., Schoene N., Guisti M.M., Moyer M.P., Magnuson B.A. (2003). Anthocyanin-rich extract from Aronia meloncarpa E induces a cell cycle block in colon cancer but not normal colonic cells. Nutr. Cancer.

[19] Xu M., Bower K.A., Wang S., Frank J.A., Chen G., Ding M., Wang S., Shi X., Ke Z., Luo J. (2010). Cyanidin-3-glucoside inhibits ethanol-induced invasion of breast cancer cells overexpressing ErbB2. Mol. Cancer.

[20] Narla G., Heath K.E., Reeves H.L., Li D., Giono L.E., Kimmelman A.C., Glucksman M.J., Narla J., Eng F.J., Chan A.M. (2001). *KLF6*, a candidate tumor suppressor gene mutated in prostate cancer. Science.

[21] Sangodkar J., Shi J., DiFeo A., Schwartz R., Bromberg R., Choudhri A., McClinch K., Hatami R., Scheer E., Kremer-Tal S. (2009). Functional role of the *KLF6* tumour suppressor gene in gastric cancer. Eur. J. Cancer.

[22] Miyaki M., Yamaguchi T., Iijima T., Funata N., Mori T. (2007). Difference in the role of loss of heterozygosity at 10p15 (*KLF6* locus) in colorectal carcinogenesis between sporadic and familial adenomatous polyposis and hereditary nonpolyposis colorectal cancer patients. Oncol. Basel.

[23] Mukai S., Hiyama T., Tanaka S., Yoshihara M., Arihiro K., Chayama K. (2007). Involvement of kruppel-like factor 6 (*KLF6*) mutation in the development of nonpolypoid colorectal carcinoma. World J. Gastroenterol..

[24] DiFeo A., Narla G., Hirshfeld J., Camacho-Vanegas O., Narla J., Rose S.L., Kalir T., Yao S., Levine A., Birrer M.J. (2006). Roles of *KLF6* and *KLF6*-SV1 in ovarian cancer progression and intraperitoneal dissemination. Clin. Cancer Res..

[25] Song J., Kim C.J., Cho Y.G., Kim S.Y., Nam S.W., Lee S.H., Yoo N.J., Lee J.Y., Park W.S. (2006). Genetic and epigenetic alterations of the *KLF6* gene in hepatocellular carcinoma. J. Gastroenterol. Hepatol..

[26] Yea S., Narla G., Zhao X. (2008). Ras promotes growth by alternative splicing-mediated inactivation of the *KLF6* tumor suppressor in hepatocellular carcinoma. Gastroenterology.

[27] Ozdemir F., Koksal M., Ozmen V., Aydin I., Buyru N. (2014). Mutations and kruppel-like factor 6 (*KLF6*) expression levels in breast cancer. Tumor Biol..

[28] Benzeno S., Narla G., Allina J., Cheng G.Z., Reeves H.L., Banck M.S., Odin J.A., Diehl J.A., Germain D., Friedman S.L. (2004). Cyclin-dependent kinase inhibition by the *KLF6* tumor suppressor protein through interaction with *Cyclin D1*. Cancer Res..

[29] Schieber A., Keller P., Carle R. (2001). Determination of phenolic acids and flavonoids of apple and pear by high-performance liquid chromatography. J. Chromatogr. A.

[30] Schuh R., Aicher W., Gaul U., Côte S., Preiss A., Maier D., Seifert E., Nauber U., Schröder C., Kemler R. (1986). A conserved family of nuclear proteins containing structural elements of the finger protein encoded by krüppel, a drosophila segmentation gene. Cell.

[31] Pearson R., Fleetwood J., Eaton S., Crossley M., Bao S. (2008). Krüppel-like transcription factors: A functional family. Int. J. Biochem. Cell Biol..

[32] Koritschoner N.P., Bocco J.L., Panzetta-Dutari G.M., Dumur C.I., Flury A., Patrito L.C. (1997). A novel human zinc finger protein that interacts with the core promoter element of a tata box-less gene. J. Biol. Chem..

[33] Reddy M.K., Alexander-Lindo R.L., Nair M.G. (2005). Relative inhibition of lipid peroxidation, cyclooxygenase enzymes, and human tumor cell proliferation by natural food colors. J. Agric. Food Chem..

[34] Chen P.N., Chu S.C., Chiou H.L., Chiang C.L., Yang S.F., Hsieh Y.S. (2005). Cyanidin 3-glucoside and peonidin 3-glucoside inhibit tumor cell growth and induce apoptosis in vitro and suppress tumor growth in vivo. Nutr. Cancer.

[35] Huang H.P., Chang Y.C., Wu C.H., Hung C.N., Wang C.J. (2011). Anthocyanin-rich mulberry extract inhibit the gastric cancer cell growth in vitro and xenograft mice by inducing signals of p38/p53 and c-jun. Food Chem..

[36] Charepalli V., Reddivari L., Radhakrishnan S., Vadde R., Agarwal R., Vanamala J.K.P. (2015). Anthocyanin-containing purple-fleshed potatoes suppress colon tumorigenesis via elimination of colon cancer stem cells. J. Nutr. Biochem..

[37] Song Y.P., Lee Y.K., Lee W.S., Park O.J., Kim Y.M. (2014). The involvement of AMPK/GSK3-beta signals in the control of metastasis and proliferation in hepato-carcinoma cells treated with anthocyanins extracted from Korea wild berry Meoru. BMC Complement. Altern. Med..

[38] Yang X., Luo E., Liu X., Han B., Yu X., Peng X. (2016). Delphinidin-3-glucoside suppresses breast carcinogenesis by inactivating the Akt/HOTAIR signaling pathway. BMC Cancer.

[39] Banerjee N., Talcott S., Safe S., Mertens-Talcott S.U. (2012). Cytotoxicity of pomegranate polyphenolics in breast cancer cells in vitro and vivo: Potential role of miRNA-27a and miRNA-155 in cell survival and inflammation. Breast Cancer Res. Treat..

[40] Wang L.S., Hecht S.S., Carmella S.G., Yu N., Larue B., Henry C., McIntyre C., Rocha C., Lechner J.F., Stoner G.D. (2009). Anthocyanins in black raspberries prevent esophageal tumors in rats. Cancer Prev. Res. (Phila).

[41] Tsai T.C., Huang H.P., Chang Y.C., Wang C.J. (2014). An anthocyanin-rich extract from hibiscus sabdariffa linnaeus inhibits *N*-nitrosomethylurea-induced leukemia in rats. J. Agric. Food Chem..

[42] Lim S., Xu J.T., Kim J., Chen T.Y., Su X.Y., Standard J., Carey E., Griffin J., Herndon B., Katz B. (2013). Role of anthocyanin-enriched purple-fleshed sweet potato p40 in colorectal cancer prevention. Mol. Nutr. Food Res..

[43] Duthie S.J., Gardner P.T., Morrice P.C., Wood S.G., Pirie L., Bestwick C.C., Milne L., Duthie G.G. (2005). DNA stability and lipid peroxidation in vitamin e-deficient rats in vivo and colon cells in vitro—Modulation by the dietary anthocyanin, cyanidin-3-glycoside. Eur. J. Nutr..

[44] Chang H., Yu B., Yu X.P., Yi L., Chen C.Y., Mi M.T., Ling W.H. (2010). Anticancer activities of an anthocyanin-rich extract from black rice against breast cancer cells in vitro and in vivo. Nutr. Cancer.

